# Problem-Drinking Across the Lifespan: Cross-Sectional Versus Longitudinal Effects Among Midlife and Older Adults

**DOI:** 10.1093/geroni/igab046.3726

**Published:** 2021-12-17

**Authors:** Annabel Kady, Yimei Li, Angelo DiBello, Matthew Lee

**Affiliations:** 1 Rutgers University, Baltimore, Maryland, United States; 2 Rutgers University, Piscataway, New Jersey, United States

## Abstract

When considering problem drinking from a lifespan-developmental perspective, an often-stated premise is that problem drinking escalates during adolescence, peaks around early young adulthood, and then declines throughout the remainder of the lifespan. However, while there is a strong empirical basis for such changes throughout adolescence and young adulthood, the notion of continued declines throughout midlife and older adulthood is less firmly established and based primarily on cross-sectional data. Thus, this study contrasted cross-sectional versus longitudinal age effects on problem-drinking changes across the lifespan, with particular focus on midlife and older adulthood. Analyses used data from a large, two-wave, U.S.-representative sample. We generated descriptive “porcupine figures” graphically depicting both cross-sectional and longitudinal age effects simultaneously, and we estimated mixed-ANOVAs to partition, test, and contrast cross-sectional versus longitudinal age effects. As expected, analyses confirmed the well-known rise and fall of problem drinking across young adulthood in both cross-sectional and longitudinal age effects. In contrast, in midlife and older adulthood, only cross-sectional age effects were consistent with the notion of continued age-related declines throughout these ages, whereas the longitudinal data showed a mixture of stability and escalation at these ages. Age-confounded cohort effects are one plausible explanation for how cross-sectional data can lead to spurious conclusions about developmental change. By potentially yielding a more accurate understanding of lifespan-developmental change in midlife and older adulthood, findings like ours could help guide lifespan-developmentally-informed interventions for midlife and older-adult problem drinkers; an objective of increasing importance in light of the ongoing aging of the U.S. population.

